# Microfluidic Based Whole-Cell Biosensors for Simultaneously On-Site Monitoring of Multiple Environmental Contaminants

**DOI:** 10.3389/fbioe.2021.622108

**Published:** 2021-03-09

**Authors:** Yiqi Cao, Baiyu Zhang, Zhiwen Zhu, Xiayin Xin, Hongjing Wu, Bing Chen

**Affiliations:** Northern Region Persistent Organic Pollution Control (NRPOP) Laboratory, Faculty of Engineering and Applied Science, Memorial University of Newfoundland, St. John’s, NL, Canada

**Keywords:** synthetic biology, environmental monitoring, microfluidic system, artificial intelligence, sensing module

## Abstract

Monitoring of environmental contaminants serves a vital role in proactive environmental management and pollution control. Research efforts have been centered on the development of robust whole-cell biosensors in recent years. However, data acquisition, multiple contaminants detection and biosafety issues limit the on-site application of such biosensors. Microfluidic system exhibits great potential to face these challenges via coupling biosensors. Here, we prospect a novel microfluidic based whole-cell biosensor (MWCB) for multiplexing monitoring of diverse contaminants, and design strategies to further increase the specificity, sensitivity and accuracy, reduce signal delay and expand shelf life of the proposed MWCB for on-site environmental applications. The development of MWCB demands multidisciplinary cooperation, and the sensing platforms are highly promising for real-world contaminants monitoring.

## Introduction

Increasing discharge of multitudinous contaminants into environments has caused detrimental impacts on the ecosystem and human health. Detecting and monitoring the distribution of contaminants is fundamental for decision making and environmental management. Sensors are considered as the most valuable tools for direct, fast, and on-site monitoring (Justino et al., 2015). In contrast to traditional physical and chemical sensors for contaminants detection, biosensors have superiorities in relatively accurate and reliable real-time detection, decreased consumption of hazardous chemicals and reagents, and cost efficiency for manufacture (Mohamed, 2020). Each biosensor is composed of a biomolecule recognition element (e.g., enzyme, antibody, or cell receptor) and a bio-transducer or an electronic unit for signal and data acquisition. A wide variety of biosensors have been developed based on the transduction principles (e.g., optical, electrochemical, colorimetric, and piezoelectrical), whose operation mechanisms and environmental applications have been extensively reviewed and compared (Long et al., 2013; Marrazza, 2014; Mehrotra, 2016; Arduini et al., 2017; Pashchenko et al., 2018). Among them, the colorimetric biosensors can rapidly respond to the target contaminants and are usually directly visible without external signal transducing equipment, hence simplifying the data acquisition process and demonstrated great potential for on-site applications.

Whole-cell biosensors have attracted increasing attention currently due to their superiorities than the enzyme or antibody based biosensors, which suffer from expensive macromolecules isolation cost, limited detection capacity, and short usage lifetime (Daunert et al., 2000; Gui et al., 2017). The sensing modules in the whole-cell biosensors can quantitatively detect a series of contaminants via expressing different signal intensities. With the development of synthetic biology, many sensing modules have been excavated for individual detection of environmental contaminants, from inorganics (e.g., heavy metals including Cu, Ag, Zn, Pb, Co, Cd, Hg, As, and Ni) (Jia et al., 2019; Mendoza et al., 2020; Wang D. et al., 2020) to organics (e.g., alkanes, aromatic hydrocarbons, and antibiotics) (Sun et al., 2017; Rebets et al., 2018; Ma et al., 2020). The colorimetric whole-cell biosensors are also developed using the reporter genes, like *lacZ* (coding β-galactosidase), *crtA* and *crtI* (coding carotenoid synthesis) (Yoshida et al., 2008), and *RFP* (coding coral red fluorescence proteins) (Chong and Ching, 2016). However, several scientific gaps (e.g., precise data acquisition, multiple contaminants detection, signal delay and biosafety issues, and shield life of the product) limit the on-site application of whole-cell biosensors.

Originated for chemically and biologically analytical measurements, the microfluidic system is emerging for high-throughput molecular screening and diagnostics (Volpatti and Yetisen, 2014; Cao et al., 2020). Recent researches demonstrated that integrating the cellular biosensors into microfluidic systems could directly display the target contaminants levels through expressing colorimetric reporters and was proven successful for monitoring heavy metals (i.e., arsenic) in water (Volpetti et al., 2017; Lu et al., 2019; Wan et al., 2019b). Such deployment enabled direct and easy data retrieving through visual inspection by USB microscopes and cell phones (Wan et al., 2019b). Further, the used device could be brought back to lab for sterilization to prevent the discharge of bacterial materials, resolving potential biosafety issues.

However, applications of the existing microfluidic systems are still limited by one-pollutant-per-time monitoring capability with a narrow spectrum of detectable contaminants. The complexity of waterborne contaminants urges the development of multiple-target detection/monitoring tools with high preciseness. Synthetic biology tools can help to create a strain that integrates multiple sensing modules to identify the presence of multiple contaminants (Xia et al., 2019). However, it is facing difficulties in avoiding crosstalk between each module and extending the cellular genetic capacity. Assimilated to a signal unit or an integrated circuit of a chip, an engineered cellular biosensor is a processing device in the microfluidic system. Through designing the distribution of each cellular biosensor and modulating the performance toward specific contaminants, accurate quantification of multiple contaminants can theoretically be achieved.

Therefore, we propose the integrated microfluidic based whole-cell biosensor (MWCB) system to achieve simultaneous identification and quantification of contaminants. Further, we prospect strategies to improve its feasibility in the field by addressing issues regarding selectivity, sensitivity and accuracy, signal delay of the analysis, and product shield life. The manufacture of a MWCB demands multidisciplinary cooperation, and its on-site applications will advance our understanding on the distribution of contaminants in the future.

## Fabricating MWCB to Display Multiple Contaminants Levels

**FIGURE 1:**
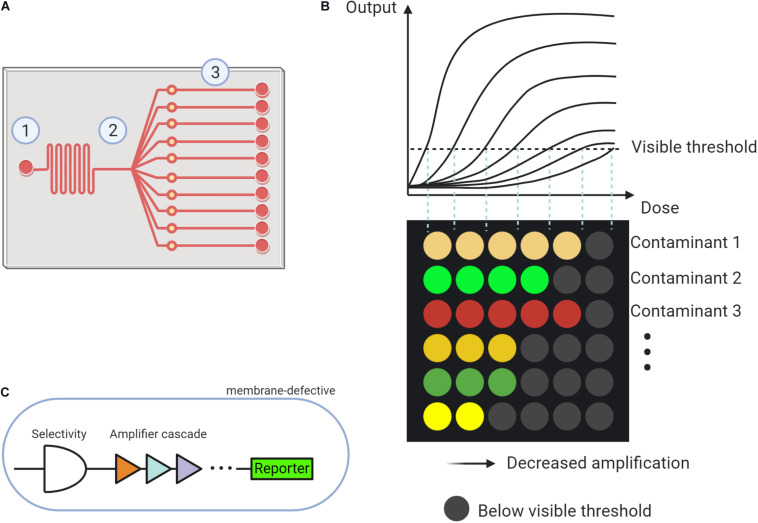
The proposed microfluidic based whole cell biosensor (MWCB). **(A)** Scheme describing the microfluidic system composing of (1) Sampling injection, (2) Partitioning, and (3) Reaction units. **(B)** MWCB for detecting types and levels of multiple contaminants, simultaneously. **(C)** Strategies for improving specificity and sensitivity and decreasing signal delay in synthetic cells of each signal processing unit.

The scheme of a microfluidic system is displayed in Figure 1A. The microfluidic system typically contains three parts: (1) sampling injection, (2) partitioning, and (3) reaction units. The design of the microfluidic system, especially the distribution of reaction parts, varied for different usages. Expanding the amounts of partitioning can simultaneously trigger multiple reactions for analysis. To encapsulate the microfluidic device, photolithographic or 3D printing techniques can be employed (Mayer et al., 2019).

Diverse inorganic (e.g., glass, silicon, and ceramics), polymeric (e.g., elastomers and thermoplastics), and emerging paper (e.g., cellulose) based materials can be used to fabricate microfluidic devices, depending on the required function, degree of the integration and applications (Nge et al., 2013). Previously studied microfluidic based biosensors majorly used the elastomer polydimethylsiloxane (PDMS) as the substrate (Volpetti et al., 2017; Wan et al., 2019b; Wang J. et al., 2020). PDMS has many superiorities for the fabrication of microfluidic biosensors including its reasonable cost, the ability for achieving rapid and easy prototyping, the capacity of enabling multiple layers design to create complex fluidics, and its function for supporting important microfluidic components (e.g., pneumatic valves and pumps). It is also gas permeable for cellular studies and optically transparent. However, there are limitations associated with PDMS like leaching of low-molecular-weight oligomers, and susceptible to non-specific adsorption and permeation by hydrophobic molecules (Berthier et al., 2012). Reaping the benefits from other materials to form hybrid substrate shows high promise to address these issues (Wang et al., 2008). In the proposed MWCB, simultaneous detection of multiple environmental contaminants demands a high chemical stability of the materials. Hence, the ideal options are hybrid PDMS or other emerging alternatives, which should be chemically stable, and appropriate for complex microfluidic design (e.g., supporting valves), cellular survive (e.g., gas permeable), and sensing (e.g., optically transparent).

In the MWCB, each whole-cell biosensor will be specifically spotted and confined (or immobilized) into one reaction unit using a microarray robot, representing each signal processing unit. Two choices can be adopted to separate each signal processing unit including (1) applying valves in the microchannels (Volpetti et al., 2017) and (2) developing multiple layers microfluidic device and placing the signal processing units on the lower layer. During the detection process, a water sample can be injected and then partitioned to the reaction units. After incubation, the colorimetric signals, proportional to the type and concentration of the contaminant in the samples, can be generated. Each row targets an individual contaminant, and detection of different contaminants are integrated into the MWCB. The increased concentration of the contaminant can be reflected by the increased number of detectable signals (i.e., signals that reach or exceed the visible threshold, ON-signals) in each row. As shown in Figure 1B, the contaminants can be differentiated by corresponding colored dots in each column, whereas their measured concentrations can be reflected in the horizontal scale. In this scenario, from the left to the right, the signal processing units in each row adopt the gradient decreased signal amplifications toward the target contaminant (Figure 1B). The range of the target contaminant level is determined and displayed based on the number of the ON-signals without the measurements of signal levels, which is beneficial for field applications. To achieve accurate and simultaneous determination of the contaminants and their levels, specificity, sensitivity and accuracy, and signal delay issues of the MWCB should be addressed. The strategies are demonstrated in the following sections.

## Specificity for Achieving Monitoring of Multiple Environmental Contaminants

To date, biosensors exhibit a wider application for heavy metals detection than organics due to their relatively higher selectivity (Gui et al., 2017). There are also existing synthetic tools used to further enhance the selectivity toward heavy metals, like mutating the binding pockets, and then employing the multi-input systems based on Boolean logic gates (Bereza-Malcolm et al., 2015). For example, the non-specific allosteric transcription ZraR modules could detect both Zn and Pb, while ZntR modules could detect Zn and Cd. Implementing AND logic for these factors could thus significantly enhance the selectivity for Zn detection (Wang et al., 2013).

The structural analogs of organic contaminants increase the difficulty in their environmental monitoring. Organic contaminants with the similar chemical structure can be monitored simultaneously. For example, tetracyclines can be monitored using the cellular biosensor of TetR-TetA regulatory-promoter system instead of specific tetracycline (Chen et al., 2017). However, sensing modules for detecting emerging contaminants, such as pharmaceuticals and personal care products (PPCP) and micro-/nano-plastics derived compounds, are limited. The importance of contaminant monitoring calls for research efforts in developing practical and effective cellular sensing modules to tailor MWCB for on-site monitoring of environmental contaminants with diverse classes.

## Improvement of Monitoring Sensitivity and Accuracy

The signal processing units in the currently proposed MWCB have ON-signal in response to the input dose higher than the visual threshold. To improve the sensitivity, lowering the detection limit toward each contaminant is indispensable. Two strategies have been developed to expand the signal dynamic range. The first one employs the hybrid σ^70^-based promoters to magnify the signal through promoter engineering (Wan et al., 2019a); whereas the second applies multi-layered cascaded transcriptional amplifiers (Figure 1C) for ultra-sensing. Such amplifier was reported with up to 5,000-fold magnification for arsenic detection with an arsenic input lower than 1.6 ppb (Wan et al., 2019b). Integrating both strategies for each signal unit design in MWCB may further expand the sensitivity to increase its applicability.

Based on the research on integrating cellular biosensors into the microfluidic system by Wan et al. (2019b), we put forward an analytical strategy with improved accuracy. In our MWCB system, the gradient decreased amplification in each line is used to sense an increased input dose. Theoretically, infinitely expanding the types and amounts of amplifications to sense each contaminant for diverse dynamic range can improve the accuracy. However, it is a great challenge for synthetic biology due to the incredibly heavy workload. Here, we propose to proportionally mix strains with different amplifications to develop multitudinous signal processing units. For example, there are two amplifiers with the ability to magnify signals by 5 and 10 folds, respectively. Through proportional combinations, the signal can be amplified by any time between 5 and 10, theoretically. Further, Artificial Intelligence (AI) technologies (e.g., machine learning and deep learning) can be promising in simulating the experimental procedures and provide optimized solutions for amplification selections and proportions of cellular mixtures (Ding et al., 2020).

## Reducing Signal Delay for Real-Time Monitoring

Signal delay, representing the time required for generating stable signals, is a major obstacle in the way of real-time operation of colorimetric whole-cell biosensors. Signal is produced when a target contaminant activates the promoter and leads to the expression of the reporter gene. During this process, three steps (i.e., contaminants entering the cells, transcription regulating, and reporter expression) can affect the response time of the whole-cell biosensors.

Strategies for reducing signal delay, therefore, can focus on three aspects. The first one is to use membrane-defective species as the host strain. The microbial outer membrane acts as the protective barrier against environmental contaminants (Vaara, 1992), causing a restricted transfer of the target compound into the cytoplasm to active the promoter-reporter modules. The second strategy is to mutate and optimize transcription regulators, which is similar to the ones proposed for the sensitivity improvement. The third strategy is to screen and select appropriate colorimetric reporter genes to decrease the response time. The production of the reporter unaffected by metabolic fluxes in the microorganism should be considered. These three strategies were verified by Chong and Ching (2016). They demonstrated that direct evolution and optimization of the transcription regulator *DmpR* (coding dimethylphenol regulatory protein) to induce expression of reporter genes *RFP* in the membrane-defective host strain *Escherichia coli*, could realize decreased response time and increased detecting limit of the whole-cell biosensors for four-nitrophenol molecules. These strategies can be adopted in the MWCB to reduce the signal delay. Research efforts should be continuously devoted to the verifications of the proposed strategies.

## Shelf Life for Facilitating the Technical Transfer

The shelf life is defined as the time from factories or labs to a monitoring site. The shelf life of MWCB, affecting by microbial storage phase and temperature, should be considered for on-site applications.

The microbes adopted for biosensing can be stored in liquid or solid phase, depending on the nature of the microfluidic systems. For the liquid phase storage, the microbial spores (e.g., *Bacillus subtilis* spores) can be stable in sensing for more than 1 month even at 80°C (Volpetti et al., 2017). Besides, for microbes that lack the ability to produce spores (e.g., *E. coli*), the 10% glycerol can be used to preserve cells’ viability (Volpetti et al., 2017). Other protective agents like trehalose, sucrose, polyvinylpyrrolidone, and polyethylene glycol can also be used for bacterial storage, which deserve future investigations. For solid phase storage, the vacuum freezing-drying methods can be used for long term bacterial storage (Li et al., 2020). Hydrogels, including alginate beads, agarose, and silica gels, can entrap prokaryotic cells and keep them hydrated and active for a month (Wan et al., 2019a).

Temperature would affect the shelf life due to its impacts on the cellular metabolism. The lower temperature (e.g., 4°C) may slow down or inhibit microbial metabolism, which leads to the high bacterial preservation capacity. Prediction of the bacterial survival can be evaluated using the Arrhenius equation to generate the appropriate shelf life (De Silvestri et al., 2018). These methods have shown great potential in the field with further investigation expected to increase the shelf life of MWCB.

## Conclusion

For on-site contaminants monitoring, it is promising to incorporate and optimize synthetic cells into the microfluidic system. The proposed MWCB can enable multiplexing monitoring of diverse contaminants and be further improved with increased selectivity, sensitivity and accuracy, and reduced signal response time toward environmental concerning contaminants. The displayed challenging works will promote multidisciplinary cooperation, especially for synthetic biology, materials, mechanical, chemical, digital printing and informatics, in the future.

## Data Availability Statement

The original contributions presented in the study are included in the article/supplementary material, further inquiries can be directed to the corresponding author.

## Author Contributions

YC conceived the idea and wrote the manuscripts. BZ supervised the topic and helped review the manuscript. ZZ, XX, HW, and BC helped review the manuscript and provide constructive suggestions. All authors have read and agreed to the published version of the manuscript.

## Conflict of Interest

The authors declare that the research was conducted in the absence of any commercial or financial relationships that could be construed as a potential conflict of interest.
